# Outcomes of Enhanced Recovery after Surgery (ERAS) in Gynecologic Oncology: A Review

**DOI:** 10.3390/curroncol29020056

**Published:** 2022-01-28

**Authors:** Steven P. Bisch, Gregg Nelson

**Affiliations:** Division of Gynecologic Oncology, Tom Baker Cancer Centre, University of Calgary, Calgary, AB T2N 4N2, Canada; gregg.nelson@albertahealthservices.ca

**Keywords:** ERAS, enhanced recovery, gynecologic oncology, opioids, cost effectiveness, complications, compliance, length of stay

## Abstract

Enhanced Recovery After Surgery (ERAS) is a global surgical quality improvement program that started in colorectal surgery and has now expanded to numerous specialties, including gynecologic oncology. ERAS guidelines comprise multidisciplinary, evidence-based recommendations in the preoperative, intraoperative, and postoperative period; these interventions broadly encompass patient education, anesthetic choice, multimodal pain control, avoidance of unnecessary drains, maintenance of nutrition, and prevention of emesis. Implementation of ERAS has been shown to be associated with improved clinical outcomes (length of hospital stay, complications, readmissions) and cost. Marx and colleagues first demonstrated the feasibility of ERAS in gynecologic oncology in 2003; since then, over 30 comparative studies and 4 guidelines have been published encompassing major gynecologic surgery, cytoreductive surgery, and vulvar/vaginal surgery. Implementation of ERAS in gynecologic oncology has been demonstrated to provide improvements in length of stay, complications, cost, opioid use, and patient satisfaction. Increased compliance with ERAS guidelines has been associated with greater improvement in outcomes.

## 1. History and Guidelines

The concept of enhanced recovery in surgery, initially labelled “fast-track surgery”, was first published by Engelman and colleagues in cardiac surgery in 1994 [1], and the first published studies of efficacy of this concept were by Kehlet in 1995 [2]. Subsequently, the Enhanced Recovery After Surgery (ERAS) Society was formed in the early 2000s by a group of European surgeons, whose mission was to improve recovery through education, research, audit, and implementation of evidence-based practice (i.e., ERAS Society guidelines). The goal of ERAS guidelines is to minimize surgical morbidity through evidence-based measures to normalize physiology in the perioperative period. Early studies were primarily focused on colorectal surgery, and it was not until 2003 that Marx and colleagues demonstrated this concept in gynecologic oncology [3]. This study implemented 10 ERAS elements in the care of patients undergoing surgery for ovarian cancer: 69 patients were treated with ERAS and were compared with 72 patients undergoing conventional care. A benefit in length of stay and a reduction in major morbidity was demonstrated [3]. Since the publication by Marx and colleagues, there have been 31 comparative studies published on ERAS in gynecologic oncology surgery, of which, 5 randomized controlled studies have been performed [4].

The first iteration of ERAS guidelines in gynecologic oncology were published in 2016 [5,6]. The initial guidelines focused on 21 recommendations in 13 domains in the preoperative and intraoperative period, and 19 recommendations in 9 domains in the postoperative period [5,6]. In the preoperative period the guidelines highlight the importance of preoperative patient counselling and education as well as optimization of patients prior to their surgery. Preoperative optimization is achieved by encouraging reduction in alcohol and tobacco, as well as by preventing unnecessary dehydration and starvation immediately prior to surgery. Prevention of common complications through thromboembolism prophylaxis, antimicrobial prophylaxis, prophylactic antiemetics, and multimodal analgesia to minimize opioid use are pillars of perioperative ERAS care. Intraoperative ERAS guidelines aim to achieve maintenance of normovolemia and normothermia, as well as avoidance of unnecessary tubes and drains [6]. Postoperatively, the same principles of normalizing physiology (i.e., minimizing the stress response of surgery) continue by optimizing oral fluid and nutritional intake, and expediting removal of intravenous lines and urinary catheters when no longer necessary. Efforts to prevent ileus are augmented by early mobilization, gut stimulation, and the utilization of multimodal non-narcotic analgesia [5].

Updated guidelines for ERAS in gynecologic oncology were published in 2019 [7]. The updated guidelines provided new evidence for previous recommendations, as well as adding recommendations for surgical site infection reduction bundles, prehabilitation, and extending recommendations to complex surgeries, such as pelvic exenteration and cytoreductive surgery with hyperthermic intraperitoneal chemotherapy (HIPEC). The updated guidelines also provided recommendations regarding patient-reported outcomes, as well as audit and reporting recommendations [7]. Guidelines covering HIPEC in more detail were published in 2020 [8,9] in collaboration with advanced gastrointestinal surgery. Finally, guidelines for vulvar and vaginal surgery were published in 2020 to address the unique requirements of recovery in these surgeries [10].

## 2. Outcomes of ERAS Implementation

### 2.1. Length of Stay

Length of stay (LOS) has been the most studied outcome during the implementation of ERAS in gynecologic oncology; in a recent meta-analysis, 27 of the 31 studies published reported outcomes for length of stay [4]. Length of stay following surgery has implications for both patient outcomes and systemic resource utilization; hospital stay constitutes a considerable portion of the cost of performing surgery in most centers. Many studies looking at length of stay in ERAS gynecologic oncology were performed using a cohort methodology with a historical (often retrospective) control cohort. Studies in this population vary in size from under 100 patients [11,12,13] to over 500 patients [14,15,16,17] and demonstrated anywhere from a 0 to 3 day reduction in median length of stay. One such study was published in 2013 by Kalogera and colleagues and examined the effect of implementing enhanced recovery pathways on a cohort of patients undergoing staging and cytoreductive surgery for gynecologic cancer (the study also assessed urogynecology procedures). This retrospective cohort study matched 241 patients undergoing enhanced recovery with 235 historical controls. This study demonstrated a 4-day reduction in average LOS for complex cytoreductive surgery (from 10.7 days pre-intervention to 6.5 days post-intervention). Additionally, a reduction in length of stay of nearly 1 day was found in the staging cohort (from 5.1 days pre-intervention to 4.2 days post-intervention) [18]. Two large cohort studies were published in 2018, demonstrating a reduction in length of stay after implementing ERAS guidelines in gynecologic oncology [14,15]. Meyer and colleagues implemented an ERAS pathway between 2014 and 2016 at a single center in the United States of America. This study of all consecutive patients undergoing open gynecologic surgery compared patients under an ERAS program to those patients receiving historical care in the six months prior to ERAS implementation. Of the 607 patients included, over 80% had a cancer diagnosis. This study found a reduction in median length of stay from 4 days pre-ERAS to 3 days following ERAS implementation [15]. The results of implementing ERAS in gynecologic oncology across two sites in Alberta, Canada, were published by Bisch and colleagues in 2018. This study of 519 patients undergoing open surgery for presumed or confirmed gynecologic malignancy demonstrated a reduction in median length of stay from 4.0 days pre-intervention to 3.0 days post-ERAS implementation. Low-complexity surgery demonstrated a reduction in median length of stay of 1 day from 3.0 days pre-ERAS to 2.0 days post-ERAS implementation. The improvement in length of stay was even more pronounced following medium/high complexity surgery with a reduction in median length of stay from 5.0 days to 3.0 days after ERAS implementation. After adjusting for age, alcohol intake, smoking, comorbidities, and procedural complexity, the mean length of stay remained statistically significant and demonstrated a 31.4% reduction in length of stay following ERAS implementation [14].

Given the promising findings of early cohort studies in gynecologic oncology, there was an increased call for randomized evidence to support the practice in this field. To date, there have been 5 randomized controlled trials performed to assess the effect of ERAS on length of stay in gynecologic oncology [13,19,20,21,22]. The first published randomized controlled trial of ERAS in gynecologic oncology was published in 2017 [20], and additional randomized studies were published in 2020 [13,19,21,22]. Although the first study in 2017 did not demonstrate a difference in length of stay (median LOS of 3 days in both arms) [20], the findings of this study were challenged due to uncertainty regarding compliance with the 9 included ERAS elements [23]. Subsequent studies utilizing formal ERAS protocols demonstrated reductions in median length of stay of 2–3 days [13,21]. The PROFAST study randomized 99 women undergoing open surgery for suspected or confirmed ovarian carcinoma to ERAS or conventional management. With 92% compliance with 15 ERAS guideline elements, this study demonstrated a reduction in median length of stay from 9 days to 7 days in the ERAS care group [13]. Another randomized controlled trial published by Ferrari and colleagues analyzed women undergoing major gynecologic surgery to ERAS (including 18 ERAS elements) or standard care. A total of 168 patients were included and approximately 60% of those patients in each arm had a final diagnosis of a gynecologic malignancy. Those patients randomized to ERAS care demonstrated a shorter length of stay (2 days) compared with the control group (4 days). This statistically significant difference was also present on subgroup analysis looking separately at open surgery (6 days versus 3 days) and laparoscopic surgery (4 days versus 2 days) [21].

A recent meta-analysis by Bisch et al. of cohort and randomized controlled trials of ERAS in gynecologic oncology, published in 2021, demonstrated a mean reduction in length of stay of 1.64 days (95% CI 1.18 to 2.10 days) compared with historical care in an analysis involving 6345 patients in 27 studies [4]. Since the publication of that meta-analysis, there have been at least two additional cohort studies in this population demonstrating significant reductions in length of stay [16,17].

### 2.2. Complications

Early skeptics of ERAS protocols cited concerns regarding increased complications as primary motivators for not adopting changes in practice. This was especially true with regard to risks surrounding respiratory events with modern fasting guidelines and early reintroduction of oral intake following surgery. A concern regarding the association of ERAS in reducing length of stay was that this would result in increased readmission for prematurely discharged patients. To address these concerns, most modern studies of ERAS implementation assess for readmission rate and many also look at complication rate following surgery. A total of 21 of the published ERAS intervention studies to date have reported on complications, with rates of complications prior to ERAS introduction ranging from 5% to 70%, depending on the population/surgery studied and the grade of complication reported [11,14,24]. The PROFAST study looked at specific complications between the ERAS and conventional management arms of the study and found no statistically significant difference in major (Grade IIIB-V) complications between the 2 arms, but noted a 10% reduction in postoperative ileus with ERAS interventions. This result was in keeping with a similar non-randomized study of patients undergoing surgery for ovarian cancer that did not find a statistically significant difference in major complication rates with ERAS implementation [11]. The study by Ferrari and colleagues found a significantly lower rate of postoperative complications in the ERAS arm (33.3%) than the standard perioperative care arm (18.3%) (*p* = 0.02). It should be noted that both the PROFAST study and the study by Gerardi and colleagues in 2008 were not powered to detect a difference in complications and had less than 50 patients per treatment arm. Meta-analysis of the 21 studies reporting complications in gynecologic oncology ERAS (including 4974 patients) demonstrated a statistically significant reduction in the odds of complication following surgery of 32% after the implementation of ERAS protocols in gynecologic oncology (OR 0.68) [4]. The odds of readmission within the first 30 days of surgery were also reduced by 20% (OR 0.80) in the ERAS cohorts compared with traditional care [4], a finding that was also identified in the randomized PROFAST study (20% in conventional management, and 6% in ERAS, *p* = 0.034) [13]. The meta-analysis did not demonstrate a significant difference in rare complications, such as anastomotic leak rates (OR 0.93), or mortality (OR 0.61) between ERAS and non-ERAS care [4]. Finally, given that gynecologic oncology patients often require time-sensitive adjuvant treatment in the form of chemotherapy and/or radiation therapy, improved recovery after surgery is essential in allowing patients to continue with intended treatment. A study by Tankou and colleagues demonstrated that ERAS-guided perioperative care was the strongest predictor of return to intended oncologic treatment (RIOT) for patients undergoing interval cytoreductive surgery [25] with 10-fold greater odds of returning to chemotherapy 28 days following operation than those patients undergoing conventional care.

### 2.3. Cost

Implementation of ERAS protocols incurs costs associated with training, audit, and additional nursing and allied health resources [14,26]. Improvements in length of stay and complications often account for the majority of cost savings seen following ERAS implementation for other surgical specialties [26]. Cost effectiveness was reported in 7 studies of ERAS in gynecologic oncology with statistically significant cost savings, with savings ranging from around 950 USD to over 8500 USD [11,14,18,27,28,29,30] (prices adjusted to 2018 USD). Meta-analysis of these studies demonstrated a statistically significant cost savings for ERAS implementation with an average savings of 2128 USD per patient (95% CI 712.23–3544.93) [4]. Data from the PROFAST randomized study secondary analyses were recently presented and demonstrated a conservative savings of 1146 EUR per patient [31]. There are significant differences in costs estimated from surgery due to the wide variety of surgery studied in this population, as well as differences in costs between public and private health care systems. Finally, a recent health economic analysis of multiple ERAS pathways, including gynecologic oncology from Alberta, Canada, showed a return on investment (ROI) ratio of 7.3 in favor of ERAS meaning that every 1 CAD invested in the program would bring 7.3 CAD in return, confirming that ERAS is a value-based surgery protocol [26].

### 2.4. Opioid Use and Pain

The ongoing opioid crisis has driven numerous interventions to attempt to curb opioid use around the time of surgery [32]. ERAS protocols have demonstrated improvements in opioid use across numerous specialties, including gynecologic oncology. Meyer and colleagues examined opioid use along with key patient-reported outcomes amongst gynecologic oncology patients and found a 72% reduction in median opioid consumption [15]. This publication also demonstrated that 16% of patients were able to achieve an opioid-free perioperative admission up to postoperative day 3, without significant differences in pain scores [15]. In addition to decreasing opioid use, the ERAS population demonstrated faster resolution of impairment in activities with earlier resolution of fatigue, and earlier return to unimpaired walking [15]. Reduction in opioid use with ERAS protocols has been well demonstrated by randomized controlled trials in gynecologic oncology; the trial by Dickson and colleagues in 2017 demonstrated a reduction in opioid use with less opioid used on postoperative day 2, and the PROFAST study demonstrated improved pain control and lower use of opioids in the ERAS group compared with the conventional management group [13]. The study by Ferrari and colleagues demonstrated a reduction in pain scores at 12 and 24 h after surgery and found that only 37% of patients in the ERAS group requested any parenteral anesthesia postoperatively, compared with 74% of patients in the standard care group. Of note is that these randomized controlled trials advocated the use of thoracic epidural or spinal analgesia as regional block for those patients with greater risk of postoperative pain, in addition to multimodal analgesia advocated for all patients within ERAS [13,20,21]. The recent study by Joshi and colleagues demonstrated a 50% reduction in opioid use following implementation of an ERAS pathway using transversus abdominis plane blocks in lieu of epidurals as part of a multimodal pain control pathway [17]. In addition to the multimodal systemic analgesia and regional anesthetics included in ERAS protocols [7], novel studies are implementing guidelines for “tiered opioid prescribing”, based on patient and surgical factors [33]. Evidence for the efficacy of these interventions was recently published by Glaser and colleagues and demonstrated a 50% reduction in opioid use with no difference in patient satisfaction when compared with historical opioid prescribing practices [33].

### 2.5. Gastrointestinal Function

A significant barrier to discharge following gynecologic oncology surgery is delayed return of normal gastrointestinal (GI) function. Delays in recovery of the GI tract are multifactorial and can, themselves, lead to respiratory, renal, and infectious complications through the sequelae of aspiration, dehydration, and malnutrition. Despite the early introduction of oral enteral intake, ERAS has been found to decrease the risk of gastrointestinal complications [34] and is associated with shortened time to first flatus and lower rates of paralytic ileus [13,18,22,35,36]. A study specifically looking at the effect of ERAS on gastrointestinal function in gynecologic oncology was published in 2018 [36]. In this study, of 376 patients undergoing gynecologic oncology surgery, Boitano and colleagues found that ileus was significantly less common in the ERAS group compared with traditional care (2.8% versus 15.7%). Notably, the decreased risk of ileus with ERAS remained significant after adjusting for patient and surgical factors with an 80% reduction in the odds of ileus (OR 0.2). Kalogera and colleagues reported on ERAS in both gynecologic oncology and pelvic organ prolapse surgery in 2013; they found that return of bowel function (defined as passage of flatus) occurred 1 day earlier in patients treated with ERAS compared with an historical control group. Conversely, this study also demonstrated increased nausea (but not vomiting) in the ERAS care group, with 55.6% of patients reporting nausea on postoperative day 2 compared with 38.5% in the historical cohort [18].

### 2.6. Patient Satisfaction and Quality of Life

Patient satisfaction is essential to the implementation of a successful and sustainable ERAS program. Initial studies assessed patient satisfaction without the benefit of validated tools [37], whereas more recent studies have demonstrated high objective satisfaction with ERAS protocols [38], as well as improvements in satisfaction scores following the implementation of ERAS protocols in gynecologic oncology [21,39]. Philp and colleagues assessed patient satisfaction with enhanced recovery in gynecologic oncology for 106 patients using the IN-PATSAT 32 questionnaire (with additional questions). Philp demonstrated high levels of patient satisfaction with enhanced recovery surgery but noted lower scores being reported for access to healthcare, as well as nursing availability [38]. More recently, Thangavel and colleagues also demonstrated high levels of satisfaction (92.2 for laparoscopy and 92.4 for laparotomy) following ERAS implementation, measured by IN-PATSAT 32 [40]. Modesitt and colleagues assessed patient satisfaction prior to and after implementation of ERAS in 145 patients undergoing major gynecologic surgery using a centralized patient satisfaction database (Press Ganey infoEDGE). They demonstrated improved pain control, improved patient perception of teamwork, and improved ability of the nursing team to keep the patient informed during the surgical process [39]. Patient satisfaction with minimally invasive surgery in gynecologic oncology was assessed by Ferraioli and colleagues, using the validated Evaluation du Vécu de l’Anésthésie Génerale (EVAN-G) questionnaire [41]. The EVAN-G questionnaire consists of 26 items assessing attention, privacy, information, pain, discomfort, and waiting time. For the 92 patients included in this study, an overall high mean score of 81.9/100 was obtained with ERAS protocol-based care [41]. The randomized controlled trial of ERAS in gynecologic oncology by Ferrari and colleagues assessed patient satisfaction using the validated Quality of Recovery-15 instrument [42]. In this study of 168 patients undergoing major gynecologic surgery patients treated with ERAS demonstrated a significantly higher satisfaction compared with those treated with standard perioperative care (123.1 versus 91 points) [21].

## 3. Effect of Compliance

The largest study of the effect of compliance with ERAS on outcomes in gynecologic oncology involved an international collaboration of hospitals utilizing the ERAS Interactive Audit System (EIAS), a central repository of patient outcomes [30]. This study analyzed 2101 patients between January 2011 and November 2017 in 10 hospitals in Canada, the United States, and Europe. After adjusting for potential confounders, this study demonstrated that every unit of increase in ERAS guideline score was associated with 8% reduction in length of stay in low-complexity surgery, with a 12% reduction in length of stay for medium-/high-complexity surgery. For every unit increase in ERAS guideline score, there was a 12% lower likelihood of complication for low-complexity surgery [30]. Iniesta and colleagues examined the effect of compliance with ERAS protocol components and found that those with greater than or equal to 80% compliance with 21 ERAS guideline elements had significantly fewer complications (38% versus 58%) and shorter median length of stay (2 versus 3 days) than those with less than 80% compliance [43]. This group determined that compliance with specific elements—avoidance of fluid overload, early mobilization, early enteral nutrition, and early urinary catheter removal—were significantly associated with fewer postoperative complications [43]. A recent secondary analysis of the PROFAST study demonstrated that increasing compliance with ERAS elements was associated with shorter length of stay, with the effect most pronounced in patients with the highest complexity surgery [44].

## 4. Effect of Surgical Approach

A significant confounding factor in the assessment of the efficacy of ERAS guidelines is the role of minimally invasive surgery (MIS). Minimally invasive surgery in gynecologic oncology encompasses laparoscope-assisted, robotically assisted, and vaginal surgery (as well as the introduction of sentinel lymph node biopsy). The first ERAS guidelines in gynecologic oncology advocated for increased utilization of MIS in 2016 [6], and because of this, the rate of MIS is occasionally discordant between pre- and post-ERAS implementation groups. Given that MIS has been independently shown to improve postoperative pain, complications, and length of stay, opponents of ERAS have argued that the observed benefit in outcomes in many studies are due to the influence of MIS alone and not the other evidence-based interventions in ERAS protocols. Although the possibility of this bias does exist in some publications in gynecologic oncology, owing to increased rates of minimally invasive surgery following ERAS implementation [12,45,46], many studies address and correct for this within their study design and analysis. The majority of published studies of ERAS in gynecologic oncology either limited their analysis to open abdominal cases [13,14,36,47,48], minimally invasive cases [19,27,49], or performed dedicated subgroup analyses [21,50] to account for differences between open and MIS cohorts. The publication by Lambaudie and colleagues in 2017 analyzed the effect of ERAS implementation in gynecologic oncology at a minimally invasive technique expert center where more than 85% of procedures were performed using MIS [50]. This study demonstrated that, without a change in the rate of MIS procedures, enhanced recovery pathways were associated with a decrease in length of stay from 3 days to 2 days for laparoscopic procedures, with an even greater benefit of ERAS demonstrated in the robotic surgery group. This study did not demonstrate a statistically significant improvement in length of stay within the open surgery group, comprising just 25 patients [50]. The study by Ferrari and colleagues provides further clarity as the rates of laparoscopy, Pfannenstiel incision, and midline incision were equivalent between the randomized groups. In this study, median length of stay was improved in the ERAS group compared with the standard practice group, and subgroup analysis by surgical access demonstrated shorter length of stay for both endoscopy (laparoscopy) (4 days in standard practice compared with 2 days for ERAS) and open approaches (6 days for standard practice and 3 days for ERAS) [21].

## 5. Effect of Disease Site

Recognizing that the field of gynecologic oncology encompasses a broad spectrum of pathologies and anatomic surgical locations, recent guidelines have been developed specifically for vulvar/vaginal surgeries, as well as for cytoreductive and upper abdominal surgeries [9,10]. The first ERAS guidelines in gynecologic oncology were based on, and were primarily relevant to, major abdominopelvic surgeries typical to endometrial, cervical, and early ovarian carcinomas [7]. Although there is now ample evidence that ERAS is beneficial in gynecologic oncology surgery, less is known about the disease-specific effects of these protocols, especially for the less-common malignancies involving the vulva and vagina. The majority of comparative ERAS studies in a recent meta-analysis have reported on major abdominopelvic surgery [4]; therefore, these provide a disproportionate amount of evidence for surgery for ovarian, uterine, and cervical pathologies. Given that the guidelines for ERAS in vulvar and vaginal surgery were published recently [10], there is hope that further supportive evidence for implementation in these rare disease sites will be available in the near future—as of yet, this is an unmet need.

## 6. Conclusions

Growing from the foundational work by Kehlet and Engelman in the 1990s, ERAS has now become the standard of care in gynecologic oncology. ERAS implementation is supported by both retrospective and level 1 evidence along with meta-evidence demonstrating efficacy in reducing length of stay, readmission, complications, opioid use, and cost in gynecologic oncology, without increasing risks of rare complications or mortality. This improvement in outcomes appears to be in addition to, and not because of, the increased uptake in minimally invasive surgery in gynecologic oncology. Future studies utilizing recently published ERAS guidelines on vulvar and vaginal surgery are needed.
